# Diagnostic Usefulness of Serum Hyaluronic Acid in Patients with SARS-CoV-2 Infection

**DOI:** 10.3390/jcm13237471

**Published:** 2024-12-08

**Authors:** Bogdan Cylwik, Kacper Gan, Marcin Kazberuk, Ewa Gruszewska, Anatol Panasiuk, Lech Chrostek

**Affiliations:** 1 Department of Paediatric Laboratory Diagnostics, Medical University of Bialystok, 15-274 Bialystok, Poland; 2 Department of Gastroenterology, Hepatology and Internal Diseases, Provincial Welded Hospital, 15-278 Bialystok, Poland; kacpergan@gmail.com (K.G.); marcin.kazberuk@gmail.com (M.K.); anatol@panasiuk.pl (A.P.); 3 Department of Biochemical Diagnostics, Medical University of Bialystok, 15-269 Bialystok, Poland; ewa.gruszewska@umb.edu.pl (E.G.); lech.chrostek@umb.edu.pl (L.C.); 4 Department of Clinical Medicine, Medical University of Bialystok, 15-269 Bialystok, Poland

**Keywords:** serum hyaluronic acid, COVID-19 patient

## Abstract

**Background/Objective:** The aim of our study is to comprehensively assess the diagnostic usefulness of serum hyaluronic acid (HA) determination in COVID-19 patients. **Methods:** The study group included 87 patients with COVID-19 disease and 45 healthy subjects. The HA concentration was measured using the immunochemical method. **Results:** The serum HA concentration was significantly higher in the COVID-19 patients before admission to hospital than that in the controls (*p* < 0.001). Differences were found in HA levels between the groups categorized according to disease severity (*p* = 002), being significantly higher in patients with critical as compared to moderate disease severity (*p* < 0.001). The HA concentration varied depending on the type of oxygen therapy (*p* = 0.004). It was significantly higher in patients on a ventilator than in those without oxygen therapy (*p* = 0.002). In patients who qualified for the steroid treatment and immunotherapy, the HA levels were significantly higher compared to those who did not qualify for such therapies (*p* = 0.043, *p* = 0.049, respectively). The HA levels were significantly higher in patients with cytokine storm compared to those without it (*p* < 0.001) and were significantly more elevated in non-survivors than in survivors (*p* < 0.001). HA had an excellent diagnostic power (AUC = 0.994) with sensitivity (83.3%) and specificity (97.8%) in identifying patients with critical disease severity and an excellent diagnostic power (AUC = 0.932) with sensitivity (88.2%) and specificity (95.6%) in identifying non-surviving patients. **Conclusions:** In summary, the results of our study indicate that HA is closely associated with severe SARS-CoV-2 infection and could be used as a novel serum biomarker to predict the risk of disease progression and as a predictor of COVID-19 mortality.

## 1. Introduction

The coronavirus disease 19 (COVID-19) pandemic caused by the novel severe acute respiratory syndrome coronavirus 2 (SARS-CoV-2) suddenly appeared at the end of December 2019 and continues to affect many people around the world [1,2]. As of June, 2024, there were approximately 776 million confirmed cases of COVID-19 and approximately 7.1 million confirmed deaths due to this disease [3]. The course of COVID-19 was asymptomatic or assumed a varied constellation of clinical symptoms, from mild to moderate and severe/critical conditions. [4]. The percentage of asymptomatic patients was estimated to range from 22% to 42% [5,6]. A small number of patients, especially severe/critical cases, had pulmonary symptoms, including severe pneumonia or pulmonary edema, leading to acute respiratory distress syndrome (ARDS) or multi-organ failure, which was associated with a poor prognosis and high mortality [7]. With the introduction of sanitary restrictions and vaccinations, the number of new cases decreased significantly. Nevertheless, the risk of new cases and reinfection still remains, even in patients who were previously asymptomatic. Five years after COVID-19 emerged, the disease pandemic continues to spread. The prevalence of post-COVID-19 that develops new or persistent symptoms that continue at least three or more months after the acute phase of SARS-CoV-2 infection is estimated at 10–30% of non-hospitalized cases and 50–80% of hospitalized patients [8,9,10]. Today, clinicians put the main focus on early risk stratification and individualized treatment of COVID-19 [11]. From the perspective of laboratory diagnostics, there is a need to search for new markers or reuse current tests for screening, clinical management, and prevention of complications, especially those that appear post-COVID-19.

Hyaluronic acid (HA) is one of the particularly attention-grabbing parameters, and it has not yet been fully tested in COVID-19 disease. HA is a high molecular weight (ranging from 10^3^ to 10^7^ kDa) polysaccharide consisting of repeating disaccharide units of D-glucuronic acid and N-acetyl-D-glucosamine that are linked via alternating β-1,4 and β-1,3 glycosidic bonds (C_14_H_21_NO_11_)_n_. The majority of HA is synthesized by three membrane-bound HA synthase enzymes (HAS1, HAS2, HAS3). The molecular weight distribution is predominantly regulated by a balance of synthesis by the HAS enzymes and catabolism by the hyaluronidases (Hyal-1 and Hyal-2). HA is retained at the cell surface as a linear polysaccharide through interactions with several binding proteins collectively termed hyaladherens, which grant HA diverse biological activities. HA is a major natural constituent of the extracellular matrix (ECM) of all tissues and is crucial for various biological and pathological processes, including cellular metabolism, water homeostasis, and inflammation and immune reactions, and becomes elevated in response to infection, inflammation, and tissue injury [12,13,14]. Although widely distributed throughout the body, HA is found in the highest concentrations in synovial fluid and connective tissue, such as the synovial membrane of the joints [15]. Within the lung, HA is a crucial component of bronchial basement membranes, bronchiolar epithelium, alveolar tissues, and the endothelial glycocalyx, and it plays an important role in pulmonary biological functions [16].

It should be emphasized that the deposition and degradation of HA are closely related to the activity of immune cells. Therefore, it is to be expected that, in inflammation, the hyaluronic acid content and serum concentration may change. The inflammatory process may be initiated by a viral infection, including SARS-CoV-2 infection. Generally, in response to inflammation, the HA content in the tissue is elevated, which may be the result of an imbalance between the production and degradation of hyaluronic acid. Viral infections are known to commonly cause respiratory illnesses, as clearly evidenced by COVID-19.

In COVID-19 disease, following transmission via respiratory droplets, the SARS-CoV-2 virus binds to multiple cell types through the angiotensin-converting enzyme 2 (ACE2) receptor-expressing cells that are located in the lung, such as epithelial cells, alveolar epithelial cells, vascular endothelial cells, and also fibroblasts and macrophages [17,18]. It is usually found that HA occurs in large amounts in the lungs and respiratory secretions of patients with severe infection. Hence, it might be suspected that hyaluronic acid may play a key role in lung pathophysiology, mainly in the process of inflammation. A small number of severe cases of COVID-19 developed acute respiratory distress syndrome (ARDS) characterized by widespread pulmonary inflammation and airway edema [19]. It was suggested that HA may promote edema because it is highly hygroscopic and has the ability to absorb water [10]. Some authors proposed that the presence of clear liquid jelly in lung alveoli may be associated with an elevated content of HA [20,21,22]. Hellman et al. were the first to confirm this suggestion and to show an accumulation of HA in the alveolar spaces of the lungs, which was correlated with the occurrence of hypoxemia and respiratory failure in critical COVID-19 patients [23].

Serum HA concentration can be elevated during synovial inflammation, as seen in rheumatoid arthritis (RA), due to increased joint production. In RA patients, serum HA levels have been shown to correlate with synovial involvement and disease activity. Elevated HA levels have also been found in some patients with severe forms of osteoarthritis (OA), progressive systemic sclerosis (PSS), and systemic lupus erythematosus (SLE) [24,25]. In addition to rheumatic diseases, HA can be raised in various liver diseases characterized by liver fibrosis and cirrhosis. It has been proposed that the determination of serum HA levels may be useful in distinguishing cirrhotic from non-cirrhotic liver to assess the degree of liver fibrosis and monitor liver function in patients with chronic hepatitis, alcoholic cirrhosis, and primary biliary cirrhosis (PBC) [26,27,28].

The aim of our study is to comprehensively assess the usefulness of serum hyaluronic acid in the diagnosis of COVID-19, taking into account various categories of patients. The patients were classified according to disease severity, lung tissue involvement, cytokine storm, concomitant diseases, vaccination, oxygen therapy, treatment, and whether they survived or not. The strength of this study is that the HA was measured with a fully automated immunochemical method commonly available and routinely used in medical diagnostic laboratories using an immunochemical analyzer by laboratory diagnosticians, contrary to other authors, who determined HA using different methods, starting from qualitative techniques (histochemistry using a direct and specific HA staining method in tissues), through semi-automated (ELISA, enzyme-linked immunosorbent assay), or fully automated quantitative methods (e.g., chemiluminescence method) [23,29,30]. The novelty and importance of this study are that we investigated the relationships between serum HA level and lung tissue involvement (CT severity score), comorbidities, vaccination level, and type of treatment.

## 2. Materials and Methods

### 2.1. Patients

The study group included 87 patients (35 females and 52 males; mean age: 59.2 years; range: 22–89) with COVID-19 disease. They were newly diagnosed and untreated at the time of admission to the Department of Gastroenterology, Hepatology and Internal Diseases with the Centre for Diagnostics and Endoscopic Treatment between 20 November 2021, and 16 March 2022. The mean duration of COVID-19 symptoms before admission was 5.8 days (range: 1–21 days). Patients who had symptoms of COVID-19 were examined for SARS-CoV-2 infection by screening laboratory tests in the hospital emergency room. Initial diagnosis of COVID-19 was made by the detection of SARS-CoV-2 antigen in a nasopharyngeal swab with Panbio^PM^ COVID-19 Ag Rapid Test Device (Abbott Rapid Diagnostics Jena GmbH, Jena, Germany). The infection was confirmed by the positive RT-PCR test for SARS-CoV-2 RNA (real-time reverse transcription polymerase chain reaction) using SARS-CoV-2 Triplex PCR kits (Astra Biotech GmbH, Berlin, Germany) with thermocycler Azure Cielo 6 (Azure Biosystems, Dublin, OH, USA). According to information obtained from patients, 29 of them (33.3%) were vaccinated and 55 were not. Among the vaccinated, 9 received 1 dose, 12 received 2 doses, and 8 received 3 doses of the vaccine.

**Figure 1:**
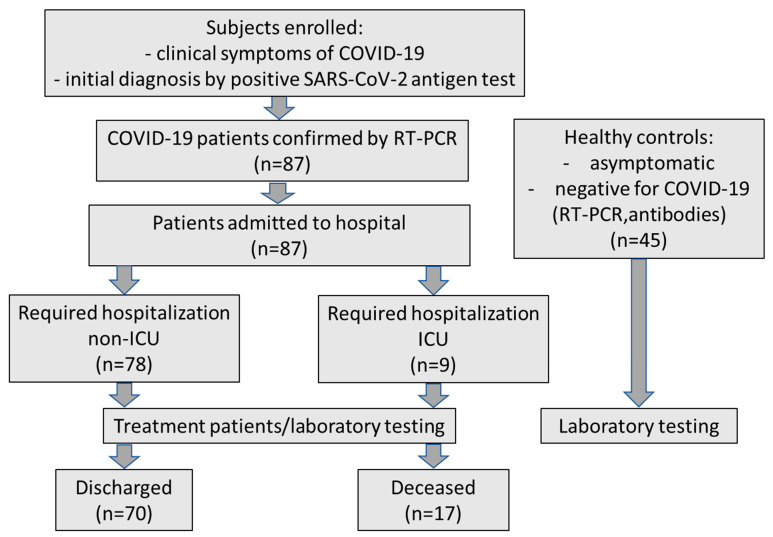
The flow chart of the recruitment of the patients in the study.

Every patient underwent laboratory tests (biochemical, hematological, coagulometric, arterial blood gas, CO-oximetric) and imaging investigations performed at the time of admission to the hospital before any treatment. During hospitalization, necessary laboratory findings were conducted at any time according to the condition till discharge or death. The control group consisted of 45 healthy volunteers (27 females and 18 males) aged 22–60 years (mean age: 31.7). They were asymptomatic, had a negative genetic test RT-PCR for SARS-CoV-2, and did not have anti-SARS-CoV-2 antibodies. The flow chart of patients’ recruitment to the study is presented in Figure 1. Written informed consent was obtained from patients and healthy subjects after explaining the nature of the study. The study was approved by the local research ethics committee for the Medical University of Bialystok (APK.002.497.2021).

### 2.2. Clinical Characteristics of Patients

Patients were classified according to disease severity, the presence of cytokine storm, concomitant diseases, and the amount of oxygen therapy required.

Taking into account the disease severity, they were categorized into 3 groups: the moderate group (58 cases), the severe group (13 cases), and the critical group (16 cases). The group with moderate disease severity included symptomatic patients (e.g., fever, cough, fatigue) with lower respiratory tract involvement but without evidence of respiratory failure, with oxygen saturation measured by pulse oximetry (SatO_2_) ≥ 94% and minor radiographic changes. In the group of patients with severe COVID-19, oxygen saturation (SatO_2_) was below 94% at a respiratory rate below 30 breaths/min, or lung infiltrates increased above 50% with respiratory failure/ARDS. Patients in the critical stage of the disease had a multi-organ dysfunction with respiratory failure/ARDS [31]. The severity of COVID-19 was evaluated on the basis of the Modified Early Warning Score (MEWS), including five parameters: systolic blood pressure, heart rate, respiratory rate, body temperature, and AVPU score (alert, verbal, pain, unconscious) (Table 1) [32]. Each of the parameters was ranked on a scale from 0 to 3 points. The total score was the sum of all components and ranged from 0 to 14. Patients with moderate COVID-19 severity had MEWS below 3 points, severe had 3–4 points, and critical had above 4 points.

Chest computed tomography severity score (CTSS) was used to quantify the severity of pulmonary involvement in COVID-19 with some triage and prognostication values as in [33]. The mean of CTSS in COVID-19 patients at baseline was 7.9 (range: 0–24). Patients were categorized based on the severity into mild disease (≤8), moderate disease (from 9 to below 15), and severe disease (15–25).

There were 28 patients with a cytokine storm (32.2%). The cytokine storm was defined by elevated levels of interleukin-6 (IL-6) above 100 µg/mL.

Hospitalized non-ICU and ICU patients with COVID-19 had chronic comorbidities (66 cases). The most common of them were hypertension (21 cases), type 2 diabetes mellitus (15 cases), and cirrhosis (8 cases).

A total of 69 patients (79.3%) required supplemental oxygen therapy. Most of them (39 cases, 44.8%) required low-flow nasal cannula oxygen therapy (oxygen flow up to 18 L/min at its concentration of 25–35%), 21 patients (24.1%) received high-flow nasal cannula oxygen therapy (flow 20–60 L/min, concentration set depending on oxygen saturation 40–100%), and 9 patients (10.3%) needed respiratory therapy (oxygen flow and its concentration depending on patient’s needs). In the group of 58 patients (66.7%) with moderate disease severity, 18 (31%) did not require oxygen administration, while 34 (58.6%) needed low-flow oxygen therapy, and in 6 sick persons (10.3%), high-flow oxygen therapy was used. Among patients with a severe form of the condition, as many as 10 (76.9%) required high-flow oxygen therapy, and only 3 (23.1%) needed low-flow oxygen therapy. In the group of patients with critical COVID-19 severity, 8 (57.1%) required connection to a respirator, 4 (28.6%) received high-flow oxygen therapy, and 2 (14.3%) received low-flow oxygen therapy.

Apart from the oxygen therapy, which is the basic method for treating respiratory failure, the patients underwent antiviral therapy (remdesivir—11 subjects), immunotherapy (tocilizumab—20 patients, barycytynib—3 patients), and steroid treatment (dexamethasone—37 patients) until improvement or death. Remdesivir (Veklury) was administered intravenously at 200 mg on the first day, followed by 100 mg daily on subsequent 4 days. Tocilizumab (RoActemra) was administered at a dose of 400 mg as a single dose via intravenous infusion to patients with a serum IL-6 greater than 100 pg/mL. Baricitinib (Olumiant) was given orally at a dose of 4 mg daily for 10 days to patients with passive oxygen therapy and present risk factors for severe disease. Dexamethasone was administered intravenously at least at a dose of 4 mg per day.

In this study, we examined 87 patients diagnosed with COVID-19, of whom 17 died in hospital (mortality rate was 19.5%), 9 were transferred to the intensive care unit (ICU) (10.3%), and 70 patients were discharged (80.5%).

### 2.3. Materials

Venous and arterial blood samples were taken twice: on admission, before any treatment (first Sample 1), and after 9 days on average (range: 4–11 days) of hospitalization (second Sample 2). The second collection took place before the patient was discharged home or transferred to the ICU. The venous blood was allowed to completely clot at room temperature. The sera were separated by centrifugation at 1500× *g* for 5 min, collected, and stored at −80 °C until analysis. Arterial blood for blood gas analysis and CO-oximetry was drawn in a standard heparinized collection tube. In addition to serum, a portion of each blood sample was collected in tubes containing EDTA-2 for hematological analysis. For coagulation tests, the blood was collected in tubes containing 3.2% sodium citrate (citrated plasma).

### 2.4. Methods

The reference ranges of normal values for all laboratory tests were provided by the reagent supplier and were not adapted to the local population.

#### 2.4.1. Determination of Hyaluronic Acid

The hyaluronic acid (HA) concentration (expected value is 23 ± 17 ng/mL) was measured by immunochemical method with WAKO reagents (Hyaluronic acid LT) adapted on the ARCHITECT ci8200 analyzer (Abbott Laboratories, Abbott Park, Chicago, IL, USA). In this method, a sample is mixed with a recombinant hyaluronic acid binding protein (rHABP), and hyaluronic acid in the sample combines specifically with HABP. In order to make an insoluble aggregate, latex particles coated by anti-HABP antibody (mouse, monoclonal) are added, and the latex binds to the above complex. As a result, the insoluble aggregate increases turbidity in the solution. The degree of turbidity of the solution can be measured optically and is proportional to the concentration of hyaluronic acid in the sample. The linearity of the method is up to 1000 ng/mL. The limit of detection of this method is 5.8 ng/mL. The total precision (CV, coefficient of variance) was not more than 5.3% at 38.5 ng/mL, 2.4% at 308.7 ng/mL, and 2.0% at 909.2 ng/mL.

#### 2.4.2. Biochemical Measurements

Biochemical assays, such as serum creatinine (expected range for men is 0.70–1.20 mg/dL and for women is 0.50–0.90 mg/dL), glucose (expected range is 70–99 mg/dL), total bilirubin (expected values for adults are up to 1.2 mg/dL), cholesterol (expected values are up to 200 mg/dL), triglycerides—TG (expected values are below 150 mg/dL), creatine kinase—CK (expected range for men is 20–200 mg/dL and for women is 20–180 mg/dL), alanine aminotransferase—ALT (expected range for men is 5–41 IU/L and for women is 5–33 IU/L), aspartate aminotransferase—AST (expected range: 5–36 IU/L), γ-glutamyltransferase—GGT (expected range for men is 10–71 IU/L and for women is 6–42 IU/L), lactate dehydrogenase—LDH (expected range for men is 135–225 mg/dL and for women is 135–214 IU/L), C-reactive protein—CRP (expected values are below 5 mg/L), and ferritin (expected range for men is 30–400 µg/L and for women is 15–150 µg/L) were measured using routine laboratory methods on a COBAS c501 analyzer (Roche/Hitachi, Tokyo, Japan). Interleukin 6—IL-6 (expected values are below 7 pg/mL) was determined by electrochemiluminescence assay on COBAS e-411 (Roche Diagnostics International Ltd, Rotkreuz, Switzerland).

#### 2.4.3. Hematological Assays

A complete blood count (CBC) was determined on the Sysmex XN-1000 analyzer (Sysmex Corporation, Singapore). The following reference values were used: RBC: 4.2–5.4 × 10^6^/µL in men and 3.5–5.2 × 10^6^/µL in women, Hb: 14–18 g/dL in men and 12–16 g/dL in women, Ht: 40–54% in men and 37–47% in women, MCV: 82–93 fL, WBC: 4–10 × 10^3^/µL, and PLT: 125–400 × 10^3^/µL.

#### 2.4.4. Coagulometric Tests

Coagulometric tests, such as prothrombin time—PT (reference range is 12–16 s), international normalized ratio—INR (reference range is 0.8–1.2), and fibrinogen (reference range is 180–400 mg/dL) were performed on an ACL TOP 300 CTS analyzer (Instrumentation Laboratory, Werfen Company, Bedford, MA, USA).

#### 2.4.5. Arterial Blood Gas (ABG) and CO-Oximetry

Arterial blood gas and CO-oximetry were performed on the ABI 90 FLEX PLUS analyzer (Radiometer Medical ApS, Copenhagen, Bronshoj, Denmark). The normal arterial blood gas and CO-oximetry were as follows: pH—7.35–7.45, PaO_2_—75–100 mmHg, PaCO_2_—32–45 mmHg, BE—from −2.5 to +2.5, and O_2_Sat—95–98%.

### 2.5. Statistics

Statistical analysis was performed using Statistica 13.3 PL (StatSoft, Cracow, Poland). The results were expressed as medians and interquartile ranges (Q1–Q3). The normality of distribution was checked by means of the Kolmogorov–Smirnov test. The analysis revealed that the distributions of Hb, RBC, MCV, PLT, and PaO_2_ follow a normal distribution. For these tests with a normal distribution, the differences between Sample 1 and controls and Sample 2 and controls were estimated by the Student’s *t*-test, whereas for the rest of the tests with a non-normal distribution, the Mann–Whitney U test was used. The differences between Sample 1 and Sample 2 were estimated by the Wilcoxon matched-pairs test. The effect of the disease severity, oxygen therapy, and Modified Early Worming Score (MEWS) on the concentration of hyaluronic acid was tested by the ANOVA rank Kruskal–Wallis test with a further post hoc analysis. For tests with a non-normal distribution, Spearman’s rank correlation coefficient was used, while for tests with a normal distribution, Pearson’s correlation test was used. The results were considered to be statistically significant when *p*-values were less than 0.05. To calculate the diagnostic accuracy of hyaluronic acid in COVID-19 patients, the area under the receiver operating characteristic (AUROC) curve was calculated. The diagnostic sensitivity, specificity, accuracy, and positive (PPV) and negative predictive values (NPV) were counted using optimal cut-offs that were determined according to the Youden method.

## 3. Results

The results of basic laboratory tests of the COVID-19 patients and controls are presented in Table 2.

On admission to the hospital, among biochemical findings, the median serum activity of ALT, AST, GGT, and LDH and the concentration of glucose and TG were significantly elevated compared to the controls (for all comparisons *p* < 0.001), whereas after hospitalization, a significant decrease in AST, LDH, and CK activity was observed (*p* = 0.005, *p* < 0.001, *p* < 0.001, respectively). The median creatinine concentration in patients with COVID-19 did not differ significantly, and the eGFR value was significantly lower compared to healthy subjects (*p* < 0.001), while after hospitalization, the creatinine level was significantly lower, and filtration was higher compared to patients before hospitalization (*p* = 0.001, *p* = 0.002, respectively). All inflammatory markers (IL-6, CRP, ferritin) were significantly increased in patients before hospitalization compared to the controls (for all comparisons *p* < 0.001), while after hospitalization, a significant decrease in CRP level (*p* < 0.001) and an increase in ferritin concentration (*p* = 0.005) were observed. IL-6 level was not significantly decreased.

Among the hematological parameters, Hb, Ht, and RBC were significantly lowered, and MCV increased in COVID-19 patients before hospitalization compared to healthy subjects (*p* < 0.001, *p* = 0.028, *p* < 0.001, *p* = 0.001, respectively), while after hospitalization of these patients, the MCV value and platelet count increased (*p* = 0.041, *p* = 0.002, respectively), and the white blood cell count significantly decreased (*p* = 0.047).

The values of all coagulation tests (PT, INR, fibrinogen) increased in patients before hospitalization compared to the controls (for all comparisons, *p* < 0.001) and did not change after hospitalization of these patients.

Among the blood gas parameters, pH and BE were significantly increased, whereas PaO_2_ and PaCO_2_ decreased in patients with SARS-CoV-2 infection before hospitalization compared to the controls (*p* < 0.001, *p* = 0.002, *p* = 0.008, *p* = 0.002, respectively). O_2_Sat in the patients’ group decreased compared to the control group, but this difference was not statistically significant.

**Figure 2:**
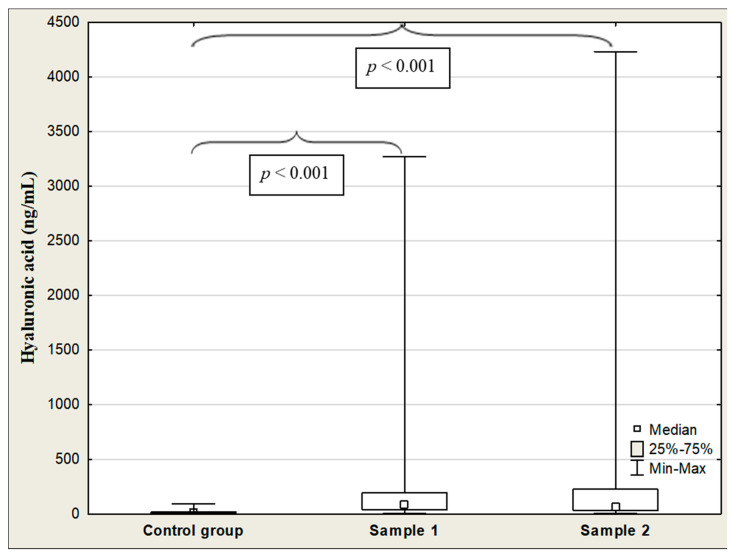
Hyaluronic acid concentrations in the control group and COVID-19 patients at admission (Sample 1) and after hospitalization (Sample 2).

The median serum HA concentrations were significantly elevated in the COVID-19 patients on admission (Sample 1) and after hospitalization (Sample 2) in comparison to the control group (83.3 vs. 13.7 ng/mL, *p* < 0.001; 67.6 vs. 13.7 ng/mL, *p* < 0.001, respectively) (Table 3, Figure 2). There was no significant difference in HA concentration between women and men in the study group (97.8 vs. 68.9 ng/mL, *p* = 0.307). Serum HA levels were not associated with age (R = 0.111, *p* = 0.307). The ANOVA rank Kruskal–Wallis test revealed differences in the HA levels depending on the patient’s age (H = 14.6, *p* = 0.007). A detailed further *post hoc* analysis showed that the HA concentrations in patients ranged from 56 to 75 years, and over 75 years were significantly higher than those in patients under 56 years of age (99.7 vs. 39.6 ng/mL, *p* = 0.002; 325.2 vs. 39.6 ng/mL, *p* = 0.012, respectively). A significant positive correlation was observed between HA levels and patients’ age (R = 0.400, *p* < 0.001). The HA levels after hospitalization were lower than those before admission to the hospital; however, no statistically significant difference was found (*p* = 0.148).

Table 4 presents the association between hyaluronic acid and laboratory tests in COVID-19 patients. Among the liver function indicators, serum HA levels were positively correlated with AST, GGT, and total bilirubin (R = 0.367, *p* < 0.001; R = 0.225, *p* = 0.040; R = 0.219, *p* = 0.043, respectively). A significant positive correlation was also observed with LDH and glucose (R = 0.414, *p* < 0.001; R = 0.267, *p* = 0.014, respectively). Among the renal function parameters, HA concentrations were positively associated with creatinine (R = 0.402, *p* = 0.007) and negatively with eGFR (R = −0.269, *p* = 0.011). Among the inflammatory indicators, significant positive correlations were observed with CRP (R = 0.410, *p* = 0.006), IL-6 (R = 0.462, *p* < 0.001), procalcitonin (R = 0.439, *p* < 0.001), ferritin (R = 0.239, *p* = 0.026), and galectin (R = 0.509, *p* = 0.000). Among the hematological parameters, HA negatively correlated with RBC (R = −0.189, *p* = 0.037), positively with MCV (R = 0.344, *p* = 0.001), and negatively with PLT (R = −0.389, *p* < 0.001). Significantly positive relationships were observed with some coagulation indexes: PT and INR (R = 0.282, *p* = 0.011 for two comparisons). Among the blood gas parameters, HA levels were negatively associated with PaO_2_ and O_2_Sat (R = −0.313, *p* = 0.005; R = −0.217, *p* = 0.047, respectively) (Table 4).

**Figure 3:**
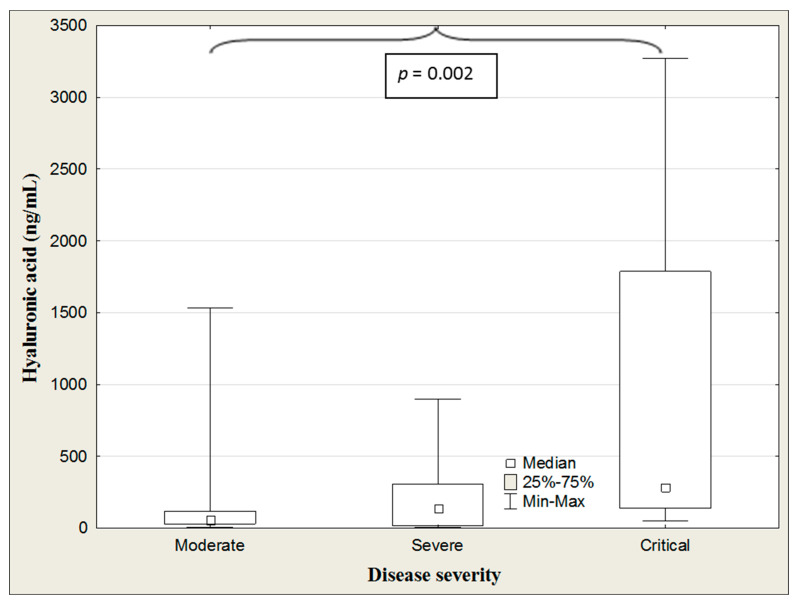
Hyaluronic acid concentrations in the COVID-19 patients according to disease severity.

The ANOVA rank Kruskal–Wallis test revealed differences in the HA concentrations between patients’ groups categorized according to disease severity (H = 20.21, *p* = 002). A detailed, further post hoc analysis showed that the HA levels in patients with a critical course of COVID-19 were significantly higher than those in moderate disease severity (*p* < 0.001). The HA concentration showed a clear upward trend with the disease severity (Table 5, Figure 3).

There was a significant correlation between the HA and the value of chest CT severity score (R = 0.465, *p* < 0.001) in patients admitted to the hospital. The values of CTSS differed according to the disease severity (H = 53.78, *p* < 0.001). It was observed that CTSS scores in the critical and severe stages of the disease were significantly higher than those in the moderate stage (16.5 vs. 2, *p* = 0.001; 14.5 vs. 2, *p* = 0.048, respectively) (Table 6).

**Figure 4:**
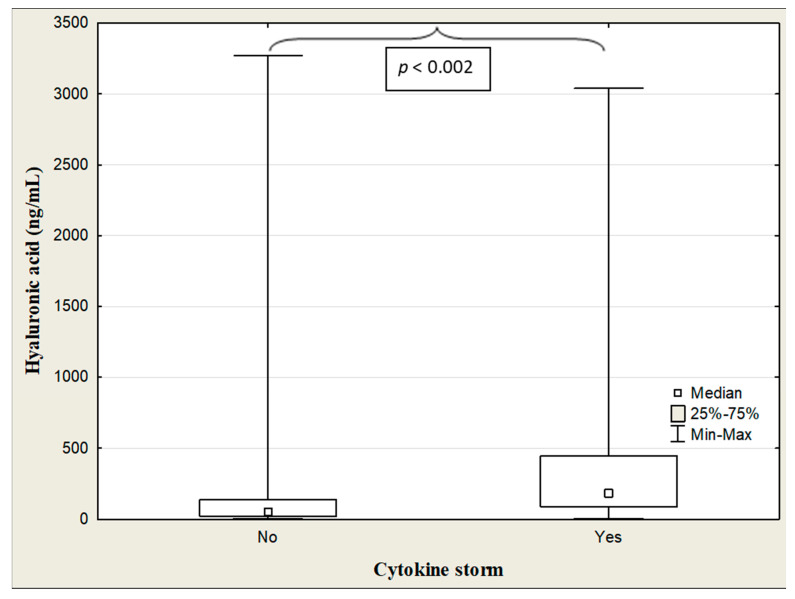
Hyaluronic acid concentrations in the COVID-19 patients with/without cytokine storm.

The HA concentration was significantly higher in admitted patients with cytokine storm compared to sick subjects without it (*p* < 0.001) (Table 7, Figure 4).

**Figure 5:**
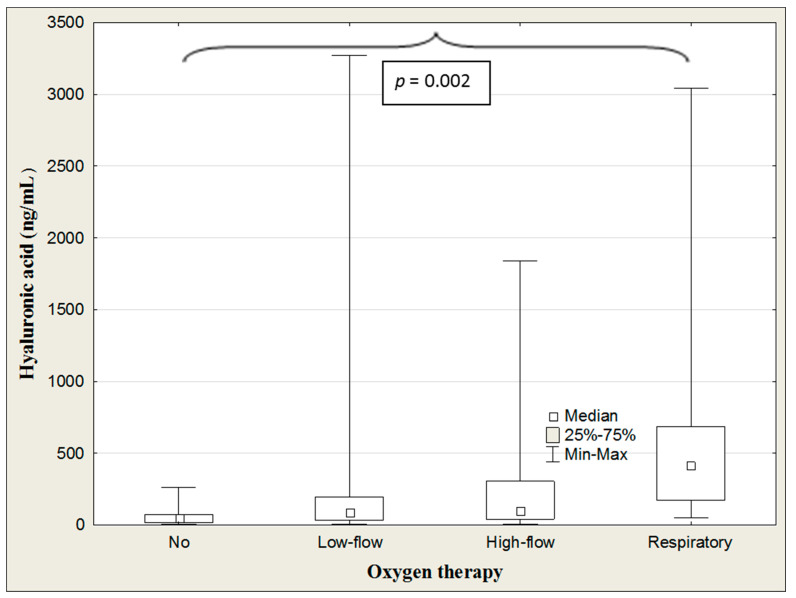
Hyaluronic acid concentrations in the COVID-19 patients with/without oxygen therapy.

The ANOVA rank Kruskal–Wallis test revealed differences in the HA levels depending on the amount of oxygen administration (H = 13.4, *p* = 0.004). The median HA concentration was significantly higher in patients connected to a ventilator than those without oxygen therapy (*p* = 0.002) (Table 8, Figure 5).

In the COVID-19 steroid treatment and immunotherapy patients, the median serum HA concentration was significantly higher compared to the patients who did not receive such therapies (107.6 vs. 55.3 ng/mL, *p* = 0.043; 129 vs. 75.3 ng/mL, *p* = 0.049, respectively) (Table 9 and Table 10). After the steroid treatment and immunotherapy, the serum HA level decreased, but the difference was not statistically significant (*p* = 0.505, *p* = 0.767, respectively) (Table 11, Table 12, Table 13 and Table 14).

There was no statistically significant HA difference between patients with concomitant chronic diseases and sick subjects without them (97.8 versus 54.1 ng/mL, *p* = 0.159). The ANOVA rank Kruskal–Wallis test revealed that there were no statistical differences in the HA levels in COVID-19 patients with various comorbidities (H = 1.414, *p* = 0.842).

**Figure 6:**
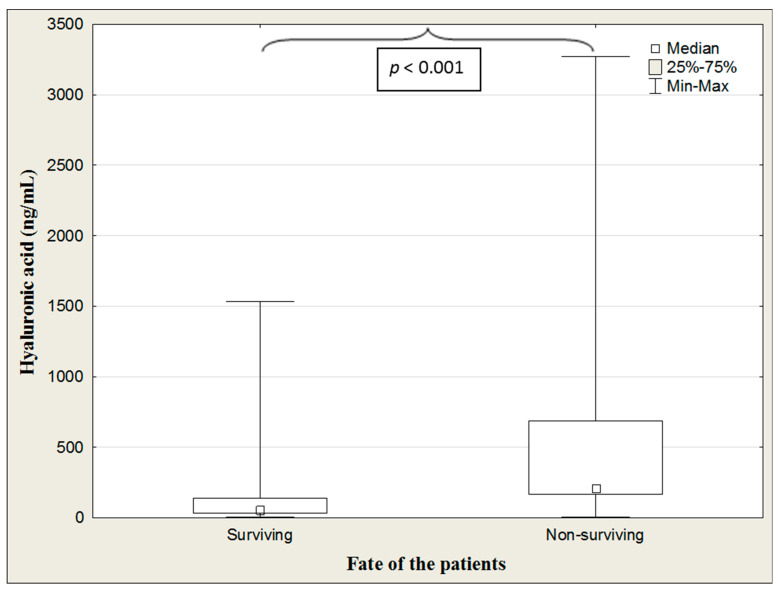
Hyaluronic acid concentrations in the surviving and non-surviving COVID-19 patients.

The level of HA was significantly higher in non-surviving than surviving patients (*p* < 0.001) (Table 15, Figure 6).

**Figure 7:**
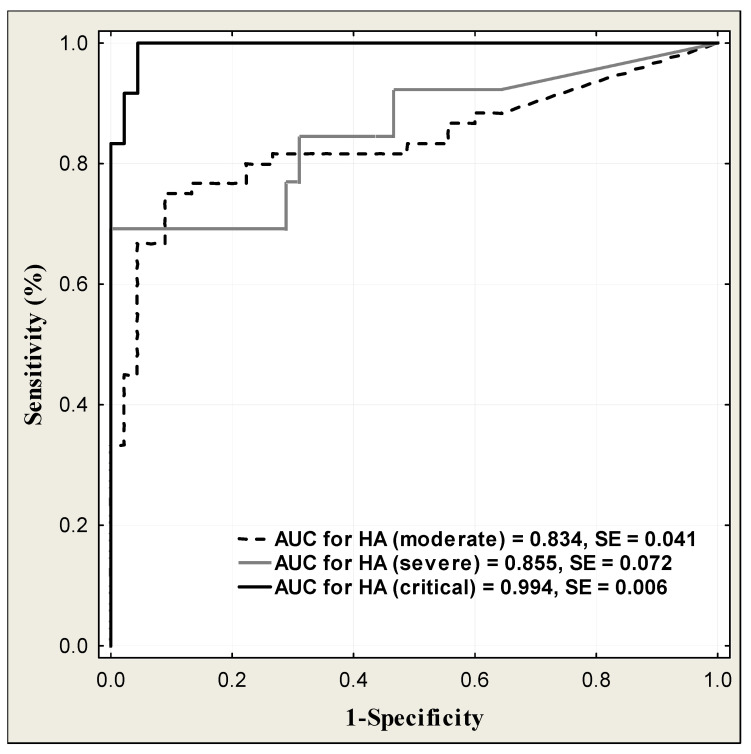
ROC curves for serum HA levels in the COVID-19 patients according to disease severity.

**Figure 8:**
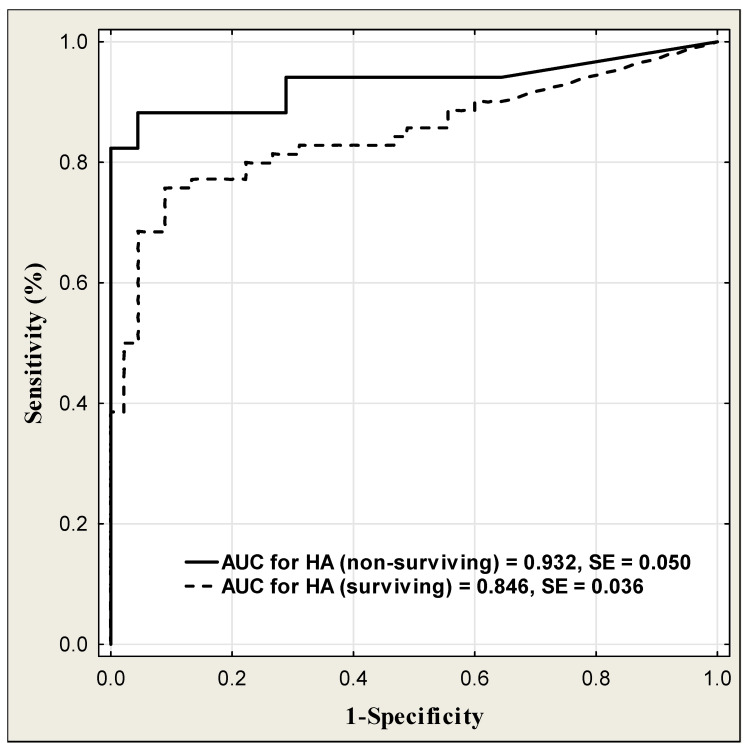
ROC curves for serum HA levels in the prediction of surviving and non-surviving COVID-19 patients.

The diagnostic power of HA in COVID-19 patients to predict disease severity is presented in Table 16 and Figure 7. Taking into account the disease severity, HA had the highest excellent diagnostic power (AUC = 0.994) in patients with critical disease severity. The diagnostic power of HA was growing up with the disease severity from moderate (AUC = 0.834), through severe (AUC = 0.855) to critical disease severity (AUC = 0.994). HA in critical condition also had the highest diagnostic sensitivity (83.3%) at the high diagnostic specificity (97.8%). The diagnostic power of HA in predicting surviving and non-surviving patients is shown in Table 17 and Figure 8. The ROC curve analysis showed that the area under curve of HA measurements was higher in non-surviving than in surviving patients (AUC = 0.932 vs. good AUC = 0.846). The HA concentration predicted the mortality of COVID-19 patients with a diagnostic sensitivity of 88.2% and specificity of 95.6%.

## 4. Discussion

The aim of our study was to evaluate the diagnostic usefulness of hyaluronic acid in patients with SARS-CoV-2 infection. Patients with COVID-19 were a mixed group consisting of women and men (mostly) aged 22–89 years, with varying disease severity and duration of symptoms ranging from 1 day to 3 weeks. Therefore, they were all divided into three groups according to COVID-19 severity. Most of them were not vaccinated and had chronic comorbidities. Some of the patients required oxygen administration. The patients underwent antiviral therapy, immunotherapy, and steroid treatment.

It was observed that serum HA concentration in COVID-19 patients was significantly increased compared to healthy individuals who were asymptomatic with a negative result of the genetic RT-PCR test for COVID-19 and no anti-SARS-CoV-2 antibodies (a six-fold increase). In our study, the HA level gradually increased with the disease severity evaluated on the basis of the MEWS score, but a statistically significant difference was only noted between the patients with critical and moderate conditions (almost a five-fold increase). Our research findings are consistent with the literature data, where the serum HA in COVID-19 patients varies significantly based on the disease severity [34,35,36]. Similarly, some authors have shown that higher concentrations of hyaluronic acid are associated with severe SARS-CoV-2 infection and, therefore, HA can be used to clearly distinguish critical from mild patients and can predict mortality in both primary infection, and as was later found, reinfection cases [29,34,35,37]. These findings suggest that serum HA could serve as a biomarker for severe acute disease and a novel indicator for predicting COVID-19 mortality and severity.

Taking into account the diagnostic power of HA, it grows with the disease severity from moderate (AUC = 0.834) through severe (AUC = 0.855) to the critical stage, reaching the excellent value (AUC = 0.994). HA in the critical stage also had the highest diagnostic sensitivity (83.3%) at the high diagnostic specificity (97.8%). The excellent diagnostic power was also observed in predicting non-surviving patients (AUC = 0.932). A comparison of HA’s diagnostic utility with other COVID-19 biomarkers revealed that ROC AUC for HA was lower than those for inflammatory markers: IL-6 (0.863 vs. 0.992), CRP (0.863 vs. 0.998), PCT (0.863 vs. 0.966), and ferritin (0.863 vs. 0.922). The HA diagnostic sensitivity and specificity were also lower than these for inflammatory indices. In the Li et al. study, the ROC analysis was also performed for some inflammatory markers, such as IL-6, CRP, and PCT, and opposite to our results, the AUC values were lower than those obtained for HA in our study [38].

The possible mechanism associated with increased HA levels and disease severity is as follows. The SARS-CoV-2 virus induces the formation of HA fragments by the pro-inflammatory cytokine IL-13, which may promote HA synthesis by the HAS1 enzyme. Accumulation of HA promotes and sustains immune cell recruitment into the lung. Next, homeostatic high molecular weight HA (HMW-HA) becomes degraded by hyaluronidases in concert with reactive oxygen species by infiltrating neutrophils. Next, low-molecular-weight HA (LMW-HA) fragments activate alveolar macrophages and induce the release of inflammatory cytokines (e.g., IL-6, IL-8, IL-10, and interferon –α), chemokines (e.g., IP-10 and MCP1), and growth factor by several cell types, including alveolar epithelial cells. Next, these pro-inflammatory LMW-HA fragments boost the effect of the cytokine storm by stimulating the release of more cytokines from immune and alveolar cells, leading to hyperinflammatory syndrome. HA fragments can directly promote endothelial barrier permeability. The edematous alveoli develop hypoxia as dysregulated HA synthesis and degradation proceed unchecked, leading to HA accumulation within respiratory fluids, alveolar collapse, and ARDS [16].

Recently, it has been shown that in patients with post-COVID-19 pulmonary fibrosis, HA concentrations were significantly elevated and associated with disease severity [39]. In another study, more than half of acute COVID-19 patients had increased serum HA and FIB-4 levels related to liver function tests, which would be evidence for the induction of liver fibrosis by multiple factors during acute COVID-19 [30]. In our study, the correlation analysis revealed relationships between HA and liver function tests and markers of liver fibrosis, such as AST, GGT, total bilirubin, ferritin, and galectin; among the COVID-19 patients, there were also those with liver cirrhosis. Thus, monitoring serum hyaluronic acid levels could be crucial in assessing the severity and progression of COVID-19 and also pulmonary and liver fibrosis.

In turn, the degree of lung involvement in COVID-19 was quantified by chest CT scan [33]. The CT severity score (CTSS) is used to quantify lung disease in COVID-19 with some triage and prognostication values [40]. Our study aimed to determine the value of CTSS in making decisions about the intensity of the treatment of respiratory failure (triage) and predicting the risk of the development of severe/critical disease in the course of COVID-19 in correlation with selected clinical parameters (prognostication). In our study, the values of CTTS gradually increased and differed depending on the disease severity, being the highest in the critical and severe COVID-19. We observed a significant correlation between CT severity score and serum hyaluronic acid. To our knowledge, no such findings have been reported in the available literature. It is worth emphasizing that the HA concentration correlates both with the disease severity evaluated by the MEWS score and with lung involvement measured by chest CT scan.

Usually, lung involvement is associated with a decrease in the oxygen saturation rate and involves the need for its administration. In our population, as many as approximately 80% of patients were provided oxygen in various amounts as low- and high-flow and respiratory therapy. We observed that with decreasing oxygen saturation, the HA level in the serum gradually increased, and it was correlated. Therefore, the HA can be a predictor not only of the disease severity but also the degree of respiratory function. The mechanisms of lung damage from COVID-19 may involve alveolar damage, endothelial injury, neutrophil extracellular traps (NETs) formation, and vascular involvement, highlighting the complexity of the disease’s impact on the respiratory system [41].

COVID-19 can trigger cytokine storm in pulmonary tissues through hyperactivation of the immune system and the uncontrolled release of cytokines [42], which, in our study, was seen in one-third of the patients. There are several mechanisms that trigger cytokine storms [43,44,45]. The SARS-CoV-2 virus activates pathogenic Th1 cells to secrete pro-inflammatory cytokines, such as granulocyte-macrophage colony-stimulating factor (GM-CSF) and interleukin-6 (IL-6). In turn, GM-CSF further activates CD14^+^CD16^+^ inflammatory monocytes to produce large quantities of IL-6, tumor necrosis factor-α (TNF-α), and other cytokines [46]. The cytokine storm in COVID-19 is characterized by a high expression of IL-6 and TNF-α. Some studies have found that patients with severe COVID-19 exhibit higher concentrations of some cytokines than patients with mild and moderate infections [47,48]. Therefore, clinicians should be able to identify patients at risk of developing severe COVID-19 as early as possible by monitoring dynamic cytokine storms. In our study, IL-6 level was significantly higher in COVID-19 patients compared to healthy individuals. The serum HA concentration in patients with cytokine storm was higher compared to those without it. The data from our study indicate that cytokines can induce the production of hyaluronic acid, suggesting a significant relationship between cytokine levels and hyaluronic acid concentration. We observed such an association between IL-6 and HA.

Most of our patients already had chronic comorbidities, such as type 2 diabetes, hypertension, and liver diseases, as the average age of the study group was approximately 60 years. It turns out that they were more susceptible to SARS-COV-2 infection. As we expected, the serum HA level was higher in patients with SARS-CoV-2 infection and comorbidities compared to the study participants without chronic complications (almost a two-fold increase), although the difference was statistically insignificant. This may be due to the wide range of results in both groups. Similar differences were also observed between chronic diseases (type 2 diabetes mellitus, hypertension, cirrhosis). The highest HA concentration was found in COVID-19 patients with type 2 diabetes (median was 140.1 ng/mL), where HA level correlated with glucose concentration. To our knowledge, there are no literature data on serum HA levels in COVID-19 patients with concomitant chronic diseases, apart from one study in which patients were divided into two subgroups according to serum HA concentrations (HA < 90 ng/mL and ≥90 ng/mL), and their numbers were compared [38]. The authors came to interesting observations that the proportions of hypertension, diabetes mellitus, and cerebrovascular disease were higher in the HA ≥ 90 ng/mL [38]. They also analyzed HA levels in COVID-19 patients complicated by diabetes mellitus and observed its elevated concentration in comparison to controls, although this difference was also not significant, like in our study.

In more than a year after the SARS-CoV-2 virus outbreak, the first vaccines were developed to protect against COVID-19 infection. This protection was not perfect, and vaccinated subjects could still develop clinical disease that requires hospitalization. In our study, almost one-third of the patients were vaccinated (they received from one to three doses of the vaccine). The serum HA concentration in vaccinated patients with COVID-19 was not specifically mentioned in the literature data. In one of the publications, it was observed that COVID-19 vaccination did not correlate with HA levels [35]. In our study, hospitalized patients with a history of SARS-CoV-2 vaccination had higher serum HA levels than those unvaccinated, but the difference was not significant. No differences were also found in the HA concentration between participants depending on the number of vaccine doses. The above authors also examined whether COVID-19 vaccination had an effect on HA expression. They divided patients into two groups according to the vaccination status below two doses and two or more doses and also found no difference in HA levels between the two groups [35].

All COVID-19 patients underwent various forms of treatment depending on individual conditions. We observed that in the group of people who were qualified for treatment, the serum HA concentration was always higher than that in those who did not require therapy. After the treatment, the HA level decreased, but the difference was not significant. We consider that an increased HA level may be an indication for the application of appropriate treatment but not for monitoring this treatment. On the other hand, some authors believe that HA may be a promising potential therapeutic target for the treatment of COVID-19 [34].

Our study group mainly included hospitalized non-ICU (approximately 90%) patients and fewer ICU patients (approximately 10%). As we expected, the HA level was significantly higher in non-surviving patients (over a three-fold increase) compared to those surviving and discharged from the hospital. In the study by Li et al., HA levels were also significantly higher in surviving patients with SARS-CoV-2 infection than in those non-surviving [35]. These researchers noted an almost two-fold increase, which was lower than that in our study. They found that patients with HA levels above 116 ng/mL had an increased risk of death. The authors concluded that HA was an independent predictor of death, and the risk of death increased by 4.435 times [35].

The main limitation of this study is the small sample size of 87 patients, which may limit the statistical validity of the study, particularly in some groups. Among the potential factors confounding our research, the presence of some comorbidities and food intake should be mentioned, which could affect the serum HA level. In our study, the patients already had chronic comorbidities, such as type 2 diabetes, hypertension, and liver diseases, as they were approximately 60 years old. These patients are more susceptible to SARS-COV-2 infection. We observed that the HA level was higher in these patients compared to those without chronic complications, but the difference was statistically insignificant. Regarding food intake, the blood for routine tests was taken in the morning and at fasting. In turn, the strength of this study was the performance of tests (on admission and after hospitalization) in the medical diagnostic laboratory of university hospitals based on routine and commonly used methods by laboratory diagnosticians. This allowed for ensuring the appropriate quality of research. There are many studies concerning laboratory tests performed on COVID-19 patients, but those on serum hyaluronic acid are relatively few, and some results of these studies are presented here for the first time.

## 5. Conclusions

In conclusion, the results of our study indicate that HA is closely associated with severe SARS-CoV-2 infection and could be used as a novel serum biomarker to predict the risk of disease progression and as a predictor of COVID-19 mortality. Physicians should consider elevated HA concentration as the risk factor in predicting the development of COVID-19 severity. High HA levels can be evidence of a cytokine storm and also an indication for treatment implementation in COVID-19 patients. In the future, given that COVID-19 continues to spread, early risk stratification and individualization treatment, especially in post-COVID-19, will be the most important. Novel therapies against the virus are needed, perhaps based on the monitoring of serum HA levels; therefore, further studies on HA utility in clinical practice are needed.

## Figures and Tables

**Table 1:** Modified Early Warning Score (MEWS) [32].

Score	3	2	1	0	1	2	3
Respiratory rate (breaths/min)	≥25	21–24		12–20			≤8
SatO_2_ (%)	≤91	92–93	94–95	≥96			
Temperature (°C)		≥39.1	38.1–39	36.1–38	35.1–36		≤35
Systolic blood pressure (mmHg)	≥220			111–219	101–110	91–100	≤90
Heart rate (bpm)	≥131	111–130	91–110	51–90	41–50		≤40
AVPU				Alert			Verbal, pain, unresponsive

Abbreviations: SatO_2_, oxygen saturation; bpm, beats per minute; AVPU, alert, verbal, pain, unresponsive.

**Table 2:** Laboratory characteristics of the study group and the controls.

Test	Sample 1 (S1)	Sample 2 (S2)	Controls (C)	Comparisons (*p*-Value)		
				S1 vs. C	S2 vs. C	S1 vs. S2
Biochemical tests						
ALT (IU/L)	30.75 15.25–56.0	35.0 22.0–62.0	10.1 7.75–14.6	<0.001 *	<0.001 *	0.074
AST (IU/L)	42.0 23.75–71.0	33.3 20.15–50.35	19.85 17.15–23.3	<0.001 *	<0.001 *	0.005 *
GGT (IU/L)	52.0 26.0–120	75.0 31.0–150	14.0 10.0–20.0	<0.001 *	<0.001 *	0.865
LDH (IU/L)	268 204–399	182.0 161.0–253.5	155.0 139.0–180.5	<0.001 *	<0.001 *	<0.001 *
CK (IU/L)	91.0 45.5–192.5	32.0 23.0–66.0	89.0 67.0–138.0	0.997	<0.001 *	<0.001 *
Total Bilirubin (mg/dL)	0.52 0.35–0.73	0.45 0.25–0.88	0.47 0.35–0.58	0.273	0.846	0.229
Glucose (mg/dL)	109 92.0–134	103.1 90.0–129.3	86.4 78.8–91.2	<0.001 *	<0.001 *	0.964
Cholesterol (mg/dL)	141.6 110.3–172.9	140.7 119.1–191.5	196.6 168.3–224.8	<0.001 *	<0.001 *	0.176
TG (mg/dL)	137.6 98.2–174.1	164.3 107.1–213.5	88.3 68.2–115.9	<0.001 *	<0.001 *	0.567
Creatinine (mg/dL)	0.85 0.71–1.24	0.77 0.60–0.95	0.86 0.76–0.94	0.506	0.009 *	0.001 *
eGFR (mL/min)	82.9 56.9–111.2	103.1 71.4–128.8	117.5 102.5–123.5	<0.001 *	0.088	0.002 *
IL-6 (pg/mL)	42.0 19.4–127	24.6 12.8–65.9	1.50 1.50–1.82	<0.001 *	<0.001 *	0.253
CRP (mg/L)	41.1 18.1–109.1	12.2 4.00–36.9	0.64 0.60–1.19	<0.001 *	<0.001 *	<0.001 *
Ferritin (µg/L)	593.5 236–1414	671.7 303.7–1245	54.5 27.3–114.5	<0.001 *	<0.001 *	0.005 *
Hematological parameters						
Hb (g/dL)	12.9 11.2–14.0	12.6 11.0–13.8	13.7 13.2–14.8	<0.001 *	<0.001 *	0.895
Ht (%)	38.4 33.4–41.3	37.6 33.5–40.8	39.1 37.1–42.35	0.028 *	0.010 *	0.570
RBC (×10^6^/µL)	4.27 3.68–4.65	4.13 3.75–4.61	4.59 4.32–4.93	<0.001 *	<0.001 *	0.895
MCV (fL)	89.2 85.5–94	89.5 85.7–93.25	85.6 83.5–88.4	0.001 *	<0.001 *	0.041 *
WBC (×10^3^/µL)	7.2 4.85–9.20	6.95 5.18–9.90	6.49 5.52–7.26	0.202	0.201	0.047 *
PLT (×10^3^/µL)	205.5 163–270	254.0 158–345	247.5 213.5–271	0.087	0.463	0.002 *
Coagulation tests						
PT (s)	13.7 12.6–16.3	14.0 13.0–16.7	12.4 12.15–13.15	<0.001 *	<0.001 *	0.850
INR	1.14 1.05–1.37	1.18 1.09–1.44	0.97 0.94–1.03	<0.001 *	<0.001 *	0.841
Fibrinogen (mg/dL)	431 308–613	315.5 179.5–413	294 256–342	<0.001 *	0.722	0.465
Arterial blood gas and CO-oximetry						
pH	7.457 7.426–7.484	7.459 7.436–7.488	7.405 7.375–7.425	<0.001 *	<0.001 *	0.468
PaO_2_ (mmHg)	83.6 64.0–107	82.6 65.7–107	97.0 93.0–101	0.008 *	0.007 *	0.317
PaCO_2_ (mmHg)	36.1 34.0–39.8	38.5 34.9–41.3	39.0 37.0–41.0	0.002 *	0.241	0.058
O_2_Sat (%)	96.8 93.0–98.5	96.7 94.9–98.1	97.0 97.0–99.0	0.204	0.075	0.839
BE (mEq/L)	2.50 −0.70–4.90	3.25 1.20–5.30	0.33 −0.90–1.15	0.002 *	<0.001 *	0.020 *

Data are presented as medians and interquartile ranges (Q1–Q3). The differences between S1 and C and S2 and C were estimated by Student’s *t*-test (for Hb, RBC, MCV, PLT, pO2) or Mann–Whitney U test (for the rest test). The differences between S1 and S2 were estimated by Wilcoxon matched-pairs test. * Significant difference at *p* < 0.05. Abbreviations: ALT, alanine aminotransferase; AST, aspartate aminotransferase; GGT, γ-glutamyltransferase; LDH, lactate dehydrogenase; CK, creatine kinase; TG, triglycerides; eGFR, estimated glomerular filtration rate; IL-6, interleukin 6; CRP, C-reactive protein; Hb, hemoglobin; Ht, hematocrit; RBC, red blood cells; MCV, mean corpuscular volume; WBC, white blood cells; PLT, platelets; PT, prothrombin time; INR, international normalized ratio; PaO_2_, partial pressure of arterial oxygen; PaCO_2_, partial pressure of arterial carbon dioxide; O_2_Sat, oxygen saturation; BE, base excess.

**Table 3:** Serum hyaluronic acid levels in the study group and the controls.

Test	Sample 1 (S1)	Sample 2 (S2)	Controls (C)	Comparisons (*p*-Value)		
				S1 vs. C	S2 vs. C	S1 vs. S2
HA	83.2 36.9–194.7	67.6 31.8–225.2	13.7 5.80–19.7	<0.001 *	<0.001 *	0.148

Data are presented as medians and interquartile ranges (Q1–Q3). The differences between S1 and C and S2 and C were estimated by Mann–Whitney U test. The differences between S1 and S2 were estimated by Wilcoxon matched-pairs test. * Significant difference at *p* < 0.05. Abbreviation: HA, hyaluronic acid.

**Table 4:** The association between hyaluronic acid and laboratory tests in COVID-19 patients.

Test	R	*p*
ALT (IU/L)	0.061	0.570
AST (IU/L)	0.367	<0.001
GGT (IU/L)	0.225	0.040
LDH (IU/L)	0.414	<0.001
CK (IU/L)	0.068	0.539
Total Bilirubin (mg/dL)	0.219	0.043
Glucose (mg/dL)	0.267	0.014
Cholesterol (mg/dL)	−0.161	0.136
TG (mg/dL)	−0.010	0.949
Creatinine (mg/dL)	0.402	0.007
eGFR (mL/min)	−0.269	0.011
IL-6 (pg/mL)	0.462	<0.001
CRP (mg/L)	0.410	0.006
Procalcitonin (ng/mL)	0.439	<0.001
Ferritin (µg/L)	0.239	0.026
Galectin	0.509	0.000
Hb (g/dL)	−0.076	0.486
Ht (%)	−0.100	0.355
RBC (×10^6^/µL)	−0.206	0.037
MCV (fL)	0.344	0.001
WBC (×10^3^/µL)	0.065	0.548
PLT (×10^3^/µL)	−0.389	<0.001
APTT (s)	−0.500	0.667
PT (s)	0.282	0.011
INR	0.282	0.011
Fibrinogen (mg/dL)	−0.06	0.594
pH	0.033	0.762
PaO_2_ (mmHg)	−0.313	0.005
PaCO_2_ (mmHg)	−0.032	0.772
O_2_Sat (%)	−0.217	0.047
BE (mEq/L)	−0.025	0.823

R, Spearman’s rank correlation coefficient; *p* < 0.05, significant correlation. Abbreviations: ALT, alanine aminotransferase; AST, aspartate aminotransferase; GGT, γ-glutamyltransferase; LDH, lactate dehydrogenase; CK, creatine kinase; TG, triglycerides; eGFR, estimated glomerular filtration rate; IL-6, interleukin 6; CRP, C-reactive protein; Hb, hemoglobin; Ht, hematocrit; RBC, red blood cells; MCV, mean corpuscular volume; WBC, white blood cells; PLT, platelets; APTT, activated partial thromboplastin time; PT, prothrombin time; INR, international normalized ratio; PaO_2_, partial pressure of arterial oxygen; PaCO_2_, partial pressure of arterial carbon dioxide; O_2_Sat, oxygen saturation; BE, base excess.

**Table 5:** Serum hyaluronic acid levels according to COVID-19 severity.

COVID-19 Severity	HA (ng/mL)
Moderate (n = 58)	57.5 30.2–119.1
Severe (n = 13)	137.8 19.1–304.8 ^1^ *p* = 0.564
Critical (n = 16)	281.1 139.9–1786.3 ^1^ *p* < 0.001 * ^2^ *p* = 0.417

Data are presented as medians and interquartile ranges (Q1–Q3). The values of HA differed between the degree of disease severity by ANOVA rank Kruskal–Wallis test (H = 20.2, *p* = 0.002) with a further post hoc analysis. * Significant difference at *p* < 0.05. ^1^ in comparison to the moderate stage. ^2^ in comparison to the severe stage. Abbreviations: HA, hyaluronic acid.

**Table 6:** The values of CTSS score in the COVID-19 patients according to disease severity.

COVID-19 Severity	CTSS
**Moderate** **(n = 58)**	2 0–10
**Severe** **(n = 13)**	14.5 11–15 ^1^ *p* = 0.048 *
**Critical** **(n = 16)**	16.5 10–20 ^1^ *p* = 0.001 *

Data are presented as medians and interquartile ranges (Q1–Q3). The values of CTSS differed between the degree of disease severity by ANOVA rank Kruskal–Wallis test (H = 53.78, *p* < 0.001) with a further post hoc analysis. * Significant difference at *p* < 0.05. ^1^ in comparison to the moderate stage. Abbreviations: CTSS, chest computed tomography severity score.

**Table 7:** Serum hyaluronic acid levels in the COVID-19 patients according to cytokine storm.

Cytokine Storm	HA (ng/mL)
**No** **(n = 59)**	56.9 20.5–136.7
**Yes** **(n = 28)**	187 87.3–446.3 *p* < 0.001

Data are presented as medians and interquartile ranges (Q1–Q3). The difference in HA level between patients with and without cytokine storm is estimated via Mann–Whitney U test. Significant difference at *p* < 0.05. Abbreviations: HA, hyaluronic acid.

**Table 8:** Serum hyaluronic acid levels in the COVID-19 patients according to oxygen therapy.

Oxygen Therapy	HA (ng/mL)
No (n = 18)	50.9 16.8–74.1
Low-flow oxygen (n = 39)	89.7 33.1–194.7 ^1^ *p* = 0.294
High-flow oxygen (n = 21)	100.3 41.8–304.8 ^1^ *p* = 0.159
Respiratory (n = 9)	417.5 172.7–686.9 ^1^ *p* = 0.002 * ^2^ *p* = 0.087 ^3^ *p* = 0.282

Data are presented as medians and interquartile ranges (Q1–Q3). The values of HA differed between the degree of disease severity by ANOVA rank Kruskal–Wallis test (H = 13.4, *p* = 0.004) with a further post hoc analysis. * Significant difference at *p* < 0.05. ^1^ in comparison to without oxygen therapy. ^2^ in comparison to low-flow oxygen therapy. ^3^ in comparison to high-flow oxygen therapy. Abbreviations: HA, hyaluronic acid.

**Table 9:** Serum hyaluronic acid levels in the COVID-19 patients according to steroid treatment on admission.

Steroid Treatment on Admission	HA (ng/mL)
**No** **(n = 34)**	55.3 33.1–100.3
**Yes** **(n = 52)**	107.6 45.3–249.0 *p* = 0.043 *

Data are presented as medians and interquartile ranges (Q1–Q3). The difference in HA level between patients with/without steroid treatment is estimated via Mann–Whitney U test. * Significant difference at *p* < 0.05. Abbreviations: HA, hyaluronic acid.

**Table 10:** Serum hyaluronic acid levels in the COVID-19 patients according to immunotherapy on admission.

Immunotherapy on Admission	HA (ng/mL)
**No** **(n = 67)**	75.3 32.8–172.7
**Yes** **(n = 19)**	129 56.9–304.8 *p* = 0.049 *

Data are presented as medians and interquartile ranges (Q1–Q3). The difference in HA level between patients with/without immunotherapy is estimated via Mann–Whitney U test. * Significant difference at *p* < 0.05. Abbreviations: HA, hyaluronic acid.

**Table 11:** Serum hyaluronic acid levels in the COVID-19 patients according to steroid treatment before and after hospitalization.

Steroid Treatment Before/After Hospitalization	HA (ng/mL)
**Before** **(n = 22)**	119.1 59.7–233.9
**After** **(n = 22)**	73.0 37.5–319.3 *p* = 0.505

Data are presented as medians and interquartile ranges (Q1–Q3). The difference in HA level before and after steroid treatment is estimated via Wilcoxon matched-pairs test. Significant difference at *p* < 0.05. Abbreviations: HA, hyaluronic acid.

**Table 12:** Differences in the serum hyaluronic acid levels in the COVID-19 patients with/without steroid treatment before and after hospitalization.

Steroid Treatment Before/After Hospitalization	HA Difference (ng/mL)
**Yes** **(n = 22)**	12.8 49.7–107.4
**No** **(n = 16)**	20.9 0–36.3 *p* = 0.722

Data are presented as medians and interquartile ranges (Q1–Q3). The difference in HA level before and after steroid treatment is estimated via Mann–Whitney U test. Significant difference at *p* < 0.05. Abbreviations: HA, hyaluronic acid.

**Table 13:** Serum hyaluronic acid levels in the COVID-19 patients according to immunotherapy before and after hospitalization.

Immunotherapy Before/After Hospitalization	HA (ng/mL)
**Before** **(n = 9)**	82.7 41.8–233.9
**After** **(n = 9)**	66.6 37.5–367.9 *p* = 0.767

Data are presented as medians and interquartile ranges (Q1–Q3). The difference in HA level before and after immunotherapy is estimated via Wilcoxon matched-pairs test. Significant difference at *p* < 0.05. Abbreviations: HA, hyaluronic acid.

**Table 14:** Differences in serum hyaluronic acid levels in the COVID-19 patients with/without immunotherapy before and after hospitalization.

Immunotherapy Before/After Hospitalization	HA Difference (ng/mL)
**Yes** **(n = 9)**	9.81 52.2–46.4
**No** **(n = 28)**	19.9 11.6–40.7 *p* = 0.746

Data are presented as medians and interquartile ranges (Q1–Q3). The difference in HA level before and after immunotherapy is estimated via Mann–Whitney U test. Significant difference at *p* < 0.05. Abbreviations: HA, hyaluronic acid.

**Table 15:** Serum hyaluronic acid levels in the surviving and non-surviving COVID-19 patients.

COVID-19 Patients	HA (ng/mL)
**Surviving** **(n = 70)**	61.1 32.8–235
**Non-surviving** **(n = 17)**	210 169–687 *p* < 0.001

Data are presented as medians and interquartile ranges (Q1–Q3). The difference in HA level between surviving and non-surviving patients is estimated via Mann–Whitney U test. Significant difference at *p* < 0.05. Abbreviations: HA, hyaluronic acid.

**Table 16:** The diagnostic power of HA in COVID-19 patients according to disease severity.

COVID-19 Severity	Cut-Off (ng/mL)	Sensitivity (%)	Specificity (%)	ACC (%)	PPV (%)	NPV (%)	AUC ± SE
Moderate (n = 58)	32.8	75.0	91.1	81.9	91.8	73.2	0.834 ± 0.041
Severe (n = 13)	99.1	69.2	100	93.1	100	91.8	0.855 ± 0.072
Critical (n = 16)	48.8	83.3	97.8	94.7	90.9	95.7	0.994 ± 0.006

ACC, diagnostic accuracy; PPV, positive predictive value; NPV, negative predictive value; AUC, area under ROC curve; SE, standard error.

**Table 17:** The diagnostic power of HA in the prediction of surviving and non-surviving COVID-19 patients.

COVID-19 Patients	Cut-off (ng/mL)	Sensitivity (%)	Specificity (%)	ACC (%)	PPV (%)	NPV (%)	AUC ± SE
Surviving (n = 70)	32.8	75.7	91.1	81.7	93.0	70.7	0.846 ± 0.036
Non-surviving (n = 17)	48.8	88.2	95.6	93.5	88.2	95.6	0.932 ± 0.050

ACC, diagnostic accuracy; PPV, positive predictive value; NPV, negative predictive value; AUC, area under ROC curve; SE, standard error.

## Data Availability

The raw data supporting the conclusions of this article will be made available by the authors upon request.
